# Pseudohypoparathyroidism Type Ib Associated with Novel Duplications in the *GNAS* Locus

**DOI:** 10.1371/journal.pone.0117691

**Published:** 2015-02-24

**Authors:** Gustavo Perez-Nanclares, Teresa Velayos, Amaya Vela, Manuel Muñoz-Torres, Luis Castaño

**Affiliations:** 1 Endocrinology and Diabetes Research Group, Hospital Universitario Cruces, BioCruces, CIBERER, CIBERDEM, UPV-EHU, Barakaldo, Basque Country, Spain; 2 Clinical Management Unit of Endocrinology and Nutrition, Hospital Universitario San Cecilio, Instituto de Investigacion Biosanitaria de Granada, Granada, Spain; IPATIMUP/Faculty of Medicine of the University of Porto, PORTUGAL

## Abstract

**Context:**

Pseudohypoparathyroidism type 1b (PHP-Ib) is characterized by renal resistance to PTH (and, sometimes, a mild resistance to TSH) and absence of any features of Albright's hereditary osteodystrophy. Patients with PHP-Ib suffer of defects in the methylation pattern of the complex *GNAS* locus. PHP-Ib can be either sporadic or inherited in an autosomal dominant pattern. Whereas familial PHP-Ib is well characterized at the molecular level, the genetic cause of sporadic PHP-Ib cases remains elusive, although some molecular mechanisms have been associated with this subtype.

**Objective:**

The aim of the study was to investigate the molecular and imprinting defects in the *GNAS* locus in two unrelated patients with PHP-Ib.

**Design:**

We have analyzed the *GNAS* locus by direct sequencing, Methylation-Specific Multiplex Ligation-dependent Probe Amplification, microsatellites, Quantitative Multiplex PCR of Short Fluorescent fragments and array-Comparative Genomic Hybridization studies in order to characterize two unrelated families with clinical features of PHP-Ib.

**Results:**

We identified two duplications in the *GNAS* region in two patients with PHP-Ib: one of them, comprising ∼320 kb, occurred ‘de novo’ in the patient, whereas the other one, of ∼179 kb in length, was inherited from the maternal allele. In both cases, no other known genetic cause was observed.

**Conclusion:**

In this article, we describe the to-our-knowledge biggest duplications reported so far in the *GNAS* region. Both are associated to PHP-Ib, one of them occurring ‘de novo’ and the other one being maternally inherited.

## Introduction

Pseudohypoparathyroidism (PHP) is a group of rare endocrine diseases characterized by hypocalcemia, hyperphosphatemia and an elevation of PTH values due to a variable resistance to this hormone in its target organs, mainly the proximal renal tubule [1]. Its exact prevalence in diverse populations is currently unknown, although it has been estimated to be 0.79/100.000 (according to Orphanet Report Series (http://www.orpha.net/consor/cgi-bin/index.php) and [2]).

Depending on the associated phenotype and biochemical features, PHP is divided in different subtypes: PHP-Ia (OMIM#103580), PHP-Ib (OMIM#603233), PHP-Ic (OMIM#612462), PHP-II (OMIM%203330) [2]. PHP type I is caused by defects at the *GNAS* locus (20q13.2–13.3), a complex cluster which generates five transcripts using alternative first exons. Between them, the alpha subunit of the heterotrimeric stimulatory G protein (Gsα) [3], which is a signaling protein essential for the actions of PTH and many other hormones (TSH, glucagon, gonadotropins,…) through the adenylyl cyclase pathway [4].

**Fig 1:**
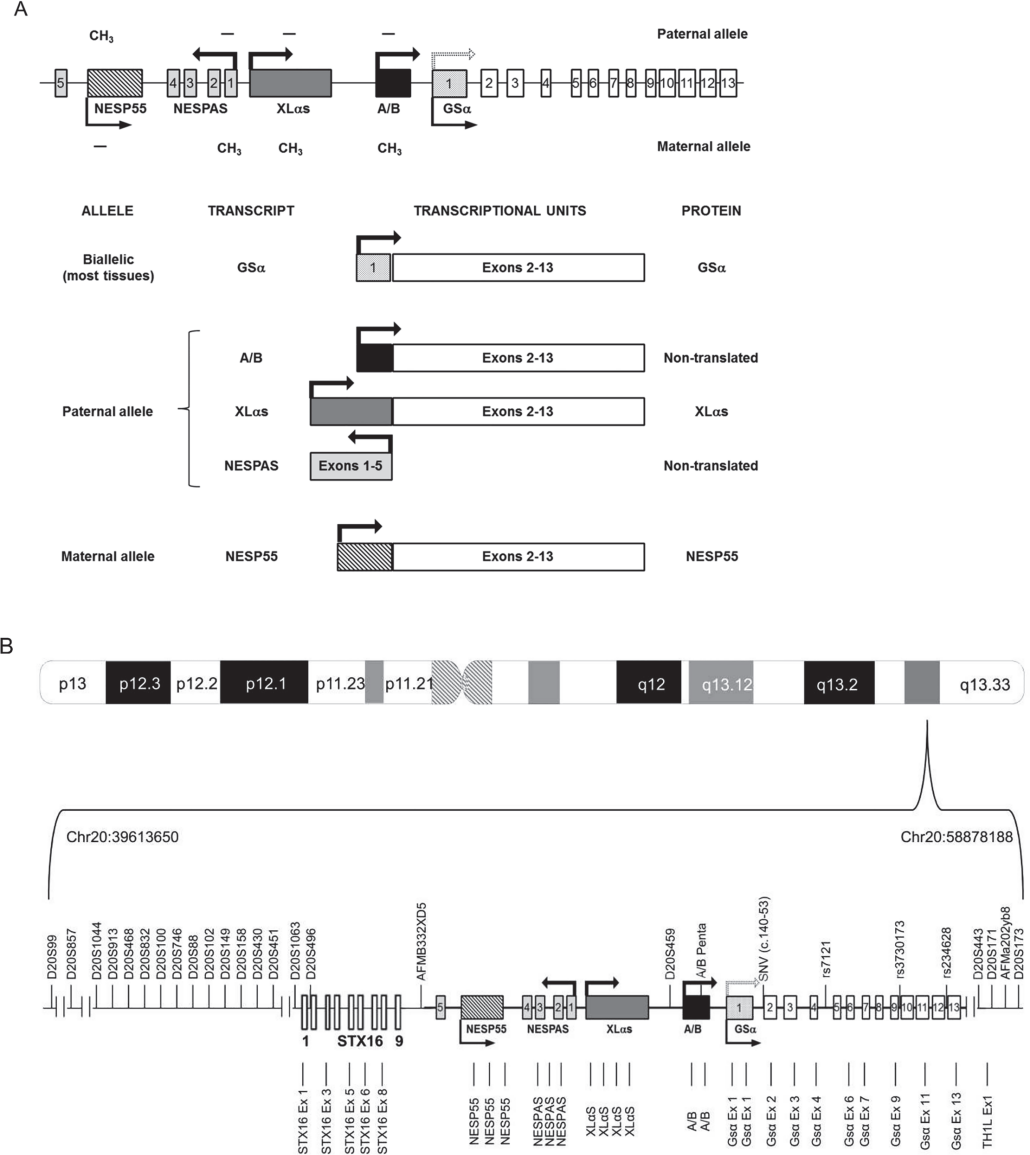
Graphic representation of the *GNAS* complex locus. (A) Alternative exons and transcripts in the region. Each first alternative exon is depicted in a different format (the scratched rectangle corresponds to NESP55, the dark grey rectangle to XLαs, the black rectangle to exon A/B and the dotted rectangle to GSα exon 1). NESPAS exons 1–5 are shown as soft grey rectangles. White rectangles represent common exons 2–13 and the continuous line represents introns. Arrows indicate the origin and sense of the transcription (dotted arrow indicates that paternal GSα is not expressed in all tissues). Methylated regions are marked with a methyl group. (B) Probes and markers used for the analysis. Polymorphic markers are depicted above, MS-MLPA probes below. See text for details. Drawings not to scale.

As mentioned above, *GNAS* is a highly complex imprinted locus that generates multiple gene products through the use of multiple promoters and first exons that splice onto a common set of downstream exons. Moreover, these transcripts can be expressed by the maternal, the paternal or both alleles as the locus is regulated by imprinting mechanisms [5] (Fig. 1A).

Patients with PHP-Ia present a characteristic phenotype called Albright’s hereditary osteodystrophy (AHO), which comprises short stature, rounded face, brachydactyly, ectopic ossifications and/or mental retardation. There is a minor response in cAMP production after exogenous PTH administration and a variable resistance to multiple hormones that act through the G protein (TSH, glucagon, gonadotropins, ACTH, GHRH…). At a molecular level, most of PHP-Ia cases are caused by haploinsufficiency because of maternally-inherited heterozygous inactivating mutations in Gsα [6]. On the contrary, when the germline mutation is inherited from the paternal allele, patients suffer of pseudo-pseudohypoparathyrodism (P-PHP, OMIM#612463). These patients present with AHO features but do not have biochemical alterations. This occurs because Gsα is predominantly transcribed from the maternal allele in the tissues where PTH, TSH, LH, FSH act (proximal renal tubule, thyroid, ovary, pituitary), so they exert their actions normally if the mutation is transmitted by the paternal allele [7].

PHP-Ib is characterized by renal resistance to PTH and absence of AHO phenotype and resistance to other hormones (although there is sometimes a mild resistance to TSH) [8]. There is also a minor cAMP production after PTH administration. PHP-Ib is due to changes in the methylation pattern of the *GNAS* locus [9]. There is a loss of methylation in the maternal exon A/B, sometimes combined with other epigenetic defects in other regions in the locus. It can be inherited in an autosomal dominant pattern (AD-PHP-Ib), although most of the cases are sporadic [2]. AD-PHP-Ib is typically characterized by an isolated loss of methylation at exon A/B, associated with microdeletions in the *STX16* gene, encoding syntaxin 16, 220 kb upstream of *GNAS* [10–12]. Less frequently, AD-PHP-Ib is caused by other microdeletions affecting NESP55 and NESPAS differentially methylated regions (DMRs) [13–15], causing broad loss of imprinting at the *GNAS* locus. Sporadic cases also show broad imprinting abnormalities at the NESPAS, XLαs and A/B DMRs, with no other changes in *cis*- or *trans*-acting elements explaining these phenomena up to now. Some PHP-Ib cases present paternal 20q disomies as the cause of the disease [16]). Moreover, an autosomal recessive form has also been hypothesized to explain the molecular mechanism of sporadic PHP-Ib in some families [17].

Recent articles have described methylation defects in some patients with PHP and AHO phenotype [18–21], an overlapping that has also been referred about the activity of the Gsα protein [22], indicating a possible molecular and clinical superposition between PHP-Ia and PHP-Ib [23].

Few genomic rearrangements have been described at the *GNAS* locus, such as terminal deletions [24] or an inversion [25], both of them related to PHP-Ia patients. To our knowledge, there is only a report describing an intralocus duplication in *GNAS* in an AD-PHP-Ib kindred [13]. In this article, we report the biggest duplications detected so far in the *GNAS* region, both associated to a PHP-Ib phenotype and arising in the maternal allele. One of them (which occurred ‘de novo’ in the patient) was associated with a complete loss of methylation at the NESPAS, XLαs and A/B DMRs, whereas the other one (present both in the index case and in her mother) was concomitant with an isolated loss of methylation in exon A/B. We did not detect any other change in the locus in either patient that could explain the differences in the methylation status, although the existence of a still unknown agent co-responsible for the (epi)phenotype cannot be discarded.

## Patients

### Case 1

The first case (GS0131) is a 16-year-old male, the only child of healthy unrelated parents, born after a normal pregnancy and a normal delivery (weight 3200 g, length: 51 cm). The mother was 26 and the father 27 years of age at the time of the pregnancy (mother’s height 157cm, father’s height 175cm). No familial history of PHP was reported. He has been properly vaccinated, with a good psychomotor development, with weight and height in the range of standard values. He has passed through an ordinary and complete puberty, and is phenotypically normal, with no symptoms of Albright’s hereditary osteodystrophy.

At age 14 years, he presented with generalized convulsions. After an altered electrocardiography was observed in the Emergency Department, he was referred to the Pediatric Cardiology Unit. All analyses were normal except for a long Q-T segment. Referred to Neuropediatrics, he was treated with different anticonvulsants, presenting a normal Magnetic Resonance Imaging (MRI). The electroencephalography showed normal but he still suffered of convulsive crisis. A scanner revealed calcifications and a biochemical evaluation showed the following values: Ca, 6.5mg/dl; P, 7.1mg/dl; PTH, 621.9pg/dl. One month later, hypocalcemia, hyperphosphatemia and PTH high values persisted (Ca, 6.1mg/dl; P, 7.8mg/dl; PTH, 884pg/dl), the patient being diagnosed of pseudohypoparathyroidism. He started oral treatment with 500 mg of calcium carbonate and 0.5 μg of calcitriol each 24 hours. Calcium and phosphate values as well as electrocardiographic alterations normalized in the first month of treatment. He is properly following the treatment and all biochemical controls have turned out to be normal, with no need of changes in the doses. He has not presented any intercurrent disease. The thyroid function analysis showed a slight hyperthyrotropinemia in the beginning, which settled down later.

The last physical evaluation (16y 5m) showed a height of 169.9cm (p25–50) and a weight of 61kg (p25-p50) (BMI: 21.1).

### Case 2

The second case (GS0155) is a 27-year-old female. She had had some episodes of right hemiparesis and a bowel volvulus in pediatric age. She was studied in the Neurology Unit after an episode of right hemiparesis, paresthesia and a visual field deficit. Imaging studies did not show any significant alteration. Biochemical analysis showed calcium values in the lower limit of normality (8.5–9.3mg/dl) and elevated PTH (200–300pg/ml), so she was diagnosed of pseudohypoparathyroidism. She did not present any symptom of Albright’s hereditary osteodystrophy (height 1.65m; weight 68.5kg; BMI: 25.1). No other hormone deficit was detected (TSH, 3μU/ml). She referred regular periods. Calcium and vitamin D supplements (both cholecalciferol and calcifediol) were prescribed, although she is not properly following the treatment.

In the last visit, she referred paresthesia in upper extremities with no calcium supplementation for a month. She brought a report with analytical levels: Ca, 8.7mg/dl; P, 3.9mg/dl; Mg, 2.4mg/dl, TSH, 4.15μU/ml; PTH, 349pg/dl; 25(OH)D, 37.2 ng/ml; urine Ca, 76mg/day; CLcr, 177ml/min; Dual-energy X-ray absorptiometry (DXA) with a lumbar T score of -0.7 SD and a femoral T score of -0.3 SD. She is clinically stable, with no symptoms of latent tetany. Biochemical analysis were performed: Ca, 8.9mg/dl; P, 3.7mg/dl; HbA1c 5.4%, PTH, 417pg/dl; 25(OH)D, 44 ng/ml.

Her mother was diagnosed at age 48 years with a multinodular toxic goitre. She was treated with I^131^ that caused iatrogenic hypothyroidism, so she was prescribed levothyroxine 125μg/day. Her past clinical history showed symptoms of generalized anxiety, being followed in the Mental Health Unit. She does not present any characteristic phenotype, with a normal physic exploration (height 1.64m; weight 70kg; BMI: 26).

## Methods

### Ethics Statement

The study was approved by the Comité Ético de Investigación Clínica (CEIC) from the Hospital Universitario Cruces. The patients and their relatives provided their written informed consent to participate in this study. In case of the minor included in the cohort, we obtained written informed consent from his guardians prior to enrolment in the study.

### Structural analysis of the GNAS locus

Peripheral blood samples from the probands and their parents were collected on EDTA tubes. DNA was extracted from whole blood using QIAamp DNA Blood Minikit (Qiagen) according to the manufacturer’s specifications. The structural analysis of the 13 Gsα exons and GNAS exon A/B was performed by polymerase chain reaction (PCR) amplification, ExoSap (USB) purification and sequencing of both strands on an ABI3130xl Genetic Analyzer (Life Technologies). Sequences were analyzed with Sequencing Analysis v.5.2 software (Life Technologies) and compared with the reference sequence (Ensembl identifier: *GNAS* locus ENSG00000087460) using SeqScape v.2.5 software (Life Technologies). Primer sequences and PCR conditions are available on request.

### Methylation analysis of the GNAS cluster by MS-MLPA

Dosage and methylation analyses of the *GNAS* locus were studied by methylation-specific multiplex ligation-dependent probe amplification (MS-MLPA) using the ME031-A2 kit (MRC-Holland) [26]. This SALSA MS-MLPA probemix is intended to provide information on deletions, duplications and abnormal methylation of sequences in the 20q13.32 *GNAS* region. The ME031-A2 *GNAS* probemix contains 27 probes specific for the complex *GNAS* locus and one for the *TH1L* gene (located 90 kb q-telomeric of *GNAS*) (Table 1, Fig. 1B). Of these, 17 are MS-MLPA probes containing a HhaI recognition site specific to the differentially methylated regions of the locus *GNAS*: XLαs, NESP55, NESPAS and *GNAS* exon A/B and one in *STX16* (located 160 kb p-telomeric of the *GNAS* locus) and in *TH1L*. Furthermore, 6 regular MLPA probes (no HhaI site) specific for *GNAS* and 4 probes for the *STX16* gene are included.

**Table 1:** Genomic location of the probes used for the MS-MLPA analysis and the 27 polymorphic markers in chromosome 20q mentioned in the text.

Probe number (MS-MLPA)	Marker/Probe	Genomic location GRCh37/hg19 coordinates
	D20S99	Chr20:39613650–39613812
	D20S857	Chr20:50108271–50108486
	D20S1044	Chr20:52452369–52452578
	D20S913	Chr20:52501053–52501308
	D20S468	Chr20:53459303–53459503
	D20S832	Chr20:53895474–53895673
	D20S100	Chr20:54314065–54314276
	D20S746	Chr20:54401983–54402234
	D20S88	Chr20:54903235–54903696
	D20S102	Chr20:54991350–54991700
	D20S149	Chr20:55514140–55514427
	D20S158	Chr20:55589222–55589604
	D20S430	Chr20:56150061–56150259
	D20S451	Chr20:56664529–56664842
	D20S1063	Chr20:57201940–57202140
P1	STX16 Ex1	Chr20:57226476–57226533
	D20S496	Chr20:57236177–57236381
P2	STX16 Ex3	Chr20:57242563–57242626
P3	STX16 Ex5	Chr20:57244321–57244372
P4	STX16 Ex6	Chr20:57245568–57245646
P5	STX16 Ex8	Chr20:57252459–57252516
	AFMB332XD5	Chr20:57257444–57257525
**P6**	**NESP55**	**Chr20:57414748–57414799**
**P7**	**NESP55**	**Chr20:57414927–57415009**
**P8**	**NESP55**	**Chr20:57415142–57415234**
**P9**	**NESPAS Ex1**	**Chr20:57425810–57425873**
**P10**	**NESPAS Ex1**	**Chr20:57425907–57426001**
**P11**	**NESPAS Int1**	**Chr20:57426013–57426065**
**P12**	**XLαs**	**Chr20:57429229–57429286**
**P13**	**XLαs**	**Chr20:57429288–57429336**
**P14**	**XLαs**	**Chr20:57430117–57430171**
**P15**	**XLαs**	**Chr20:57430201–57430258**
	D20S459	Chr20:57439859–57440088
**P16**	**A/B**	**Chr20:57464100–57464159**
	A/B Pentan	Chr20:57464278–57464282
**P17**	**A/B**	**Chr20:57464367–57464424**
P18	GSα Ex1	Chr20:57466769–57466826
P19	GSα Ex1	Chr20:57466829–57466892
	SNV (c.140–53)	Chr20: 57470614
P20	GSα Ex2	Chr20:57470713–57470779
P21	GSα Ex3	Chr20:57474005–57474058
P22	GSα Ex4	Chr20:57478577–57478646
	rs7121	Chr20:57478807
P23	GSα Ex6	Chr20:57480450–57480516
P24	GSα Ex7	Chr20:57484181–57484241
P25	GSα Ex9	Chr20:57484583–57484643
	rs3730173	Chr20:57484934
P26	GSα Ex11	Chr20:57485057–57485131
	rs234628	Chr20:57485611
P27	GSα Ex13	Chr20:57485737–57485800
	D20S443	Chr20:57495457–57495600
P28	TH1L Ex1	Chr20:57556345–57556400
	D20S171	Chr20:57808030–57808168
	AFMa202yb9	Chr20:57975178–57975420
	D20S173	Chr20:58878008–58878188

**Fig 2:**
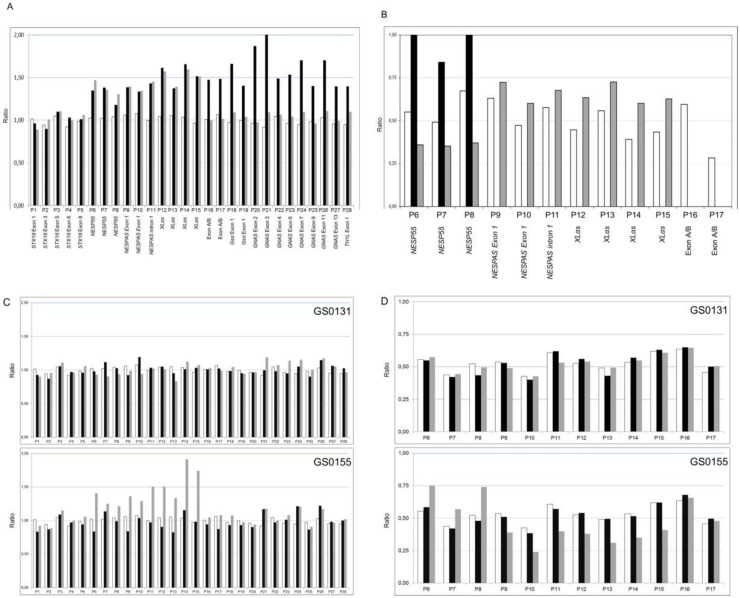
Dosage and methylation analysis of the *GNAS* locus. In all cases, on the X-axis, MS-MLPA probes for copy number determination (P1 to P28) or methylation analysis (P6 to P17) are shown according to their chromosome location (detailed in Table 1). On the Y-axis, the final probe ratio is shown. (A) Dosage analysis of the *GNAS* locus in GS0131 (black bars) and GS0155 (grey bars) compared against the average of four controls (white bars). (B) Methylation analysis of the *GNAS* locus in GS0131 (black bars) and GS0155 (grey bars) compared against the average of four controls (white bars). GS0131 shows complete loss of methylation at NESPAS, XLαs and exon A/B, and gain of methylation at NESP55, whereas GS0155 shows complete loss of methylation at A/B, partial gain at XLαs and NESPAS and partial loss at NESP55. (C) Dosage analysis of the *GNAS* locus in GS0131 parents (upper panel) and GS0155 parents (lower panel) compared against the average of four controls. In all cases, black bars represent the father, grey bars represent the mother and white bars represent the controls. Mother of GS0155 presents the same duplication as her daughter, whereas absence of alterations in GS0131 parents demonstrates its ‘de novo’ origin. (D) Methylation analysis of the *GNAS* locus in GS0131 parents (upper panel) and GS0155 parents (lower panel) compared against the average of four controls. In all cases, black bars represent the father, grey bars represent the mother and white bars represent the controls. There is not any methylation alteration in the parents of GS0131. Mother of GS0155 presents a partial loss of methylation in XLαs (P12-P15) and NESPAS (P9-P11), and a partial gain of methylation in NESP55 (P6-P8), exon A/B not being affected. See text for details.

Genomic coordinates (according to GRCh37/hg19 build) of the 28 probes used for the MS-MLPA analysis and the 27 polymorphic markers in chromosome 20q. They are situated in the table according to their chromosomal location. In the case of the MS-MLPA probes, “Probe numbers” indicate the reference used in Fig. 2. Probes highlighted in bold (P6 to P17) are used to identify methylation aberrations in the locus.

The protocol was implemented following the manufacturer's recommendations. Analysis of the MS-MLPA PCR products was performed on an ABI3130xl genetic analyzer using GeneMapper v4.0 software (Life Technologies). Each MS-MLPA reaction generates two samples. For copy number analysis, data generated by the undigested sample was intra-normalized by dividing the peak area of each amplification product by the total area of the reference probes only. The ratios were then obtained by dividing the intra-normalized probe ratio in a sample by the average intra-normalized probe ratio of all reference runs. For methylation analysis (digested sample), the intra-normalized peak area of each MS-MLPA probe from the digested sample was divided by the value obtained for the undigested sample.

### Analysis of polymorphic markers

To characterize chromosome 20q and rule out any uniparental disomy (UPD) at this locus, twenty three polymorphic markers (*D20S99*, *D20S857*, *D20S1044*, *D20S913*, *D20S468*, *D20S832*, *D20S100*, *D20S746*, *D20S88*, *D20S102*, *D20S149*, *D20S158*, *D20S430*, *D20S451*, *D20S1063*, *D20S496*, *AFMB332XD5*, *D20S459*, *D20S443*, *D20S171*, AFMa202yb9, *D20S173* and exon A/B pentanucleotide repeat) were typed by fluorescent PCR and electrophoresis on an ABI3130xl Genetic Analyzer using GeneMapper v.4.0 software (Life Technologies) (primers and conditions are available on request). Further, four single nucleotide variations (SNV) were also typed and used as markers during the structural analysis of Gsα (c.140–53, rs7121, rs3730173 and rs234628). The chromosomal location of all these markers is indicated in Table 1 and Fig. 1B.

### Whole genome and 20q13-ter Comparative Genomic Hybridization (CGH) arrays

Detection of gene copy number was performed by array-Comparative Genomic Hybridization (a-CGH) experiments. Whole genome analysis was conducted using a human whole genome 400k array (SurePrint G3 Human CGH microarray 2x400, AMADID 021850, Agilent Technologies, Santa Clara, CA), following standard and manufacturer’s protocol [27]. For the analysis of the region chr20:47897125–62435965, the array was designed using Agilent’s e-array tool (Agilent Technologies) following the manufacturer’s instruction (https://earray.chem.agilent.com/). In both cases, healthy male and female donor samples were used as hybridization controls.

Microarray data were extracted and visualized using Feature Extraction software v10.7 and Agilent Genomic Workbench v5.0 (Agilent Technologies). Copy number altered regions were analyzed using ADAM-2 (set as 6) statistic provided by DNA Analytics, with a minimum number of 5 consecutive probes (the analysis resolution, thus, is approximately 25 kb for most of the regions for the whole genome array and 400 bp for the custom array). Genomic build hg19 was used for the experiments.

### STX16 deletions

All samples from both families were screened for the 3-kb and 4.4-kb *STX16* deletions by PCR and agarose gel electrophoresis followed by ethidium bromide staining, as previously described [10, 11], with slight modifications in the PCR conditions.

### Determination of the parental origin of the duplication in family GS0131

D20S443, D20S459 and D20S1063 microsatellites, located inside the duplicated region (chr20:57381447–57701869), were genotyped by fluorescent PCR and capillary electrophoresis in order to study the inheritance of the alleles. Only D20S459 turned out to be informative (see Table 2). Quantitative multiplex PCR of short fluorescent fragments (QMPSF) was subsequently carried out for this marker together with amplicons from *AIRE* (21q22), *HNF1A* (12q24) and/or *HNF1B* (17q12) genes in a FAM-labeled multiplex PCR under semiquantitative conditions. Resulting PCR products were analyzed on an ABI3130xl Genetic Analyzer using GeneMapper v.4.0 software (Life Technologies). Data analysis was performed in Excel (Microsoft Office 2007).

**Table 2:** Genotyping results of the 27 polymorphic markers at 20q in both families.

	GS0131							GS0155						
Marker/Probe	Index		Mother		Father		Result	Index		Mother		Father		Result
D20S99	161	159	159	161	161	161	Excludes isodisomy	161	161	161	161	161	165	Excludes paternal heterodisomy
D20S857	208	208	208	216	208	214	Excludes heterodisomy	**198**	**216**	**216**	**216**	**198**	**212**	**Informative**
D20S1044	212	212	212	212	212	212	Uninformative	212	212	212	212	212	212	Uninformative
D20S913	**255**	**257**	**257**	**257**	**255**	**255**	**Informative**	255	255	255	257	255	265	Excludes heterodisomy
D20S468	207	207	207	207	207	201	Excludes paternal heterodisomy	208	192	192	208	208	202	Excludes isodisomy
D20S832	210	210	210	210	210	212	Excludes paternal heterodisomy	212	210	210	210	212	210	Excludes isodisomy
D20S100	203	207	207	203	203	203	Excludes isodisomy	**197**	**205**	**205**	**207**	**197**	**207**	**Informative**
D20S746	253	253	253	253	253	253	Uninformative	253	253	253	253	253	253	Uninformative
D20S88	**445**	**431**	**431**	**443**	**445**	**441**	**Informative**	**441**	**437**	**437**	**429**	**441**	**433**	**Informative**
D20S102	**180**	**182**	**182**	**182**	**180**	**180**	**Informative**	180	180	180	180	180	182	Excludes paternal heterodisomy
D20S149	**296**	**288**	**288**	**308**	**296**	**280**	**Informative**	**288**	**296**	**296**	**284**	**288**	**288**	**Informative**
D20S158	**388**	**392**	**392**	**392**	**388**	**382**	**Informative**	378	382	382	386	378	382	Excludes isodisomy
D20S430	**204**	**224**	**224**	**230**	**204**	**204**	**Informative**	204	204	204	216	204	208	Excludes heterodisomy
D20S451	**314**	**304**	**304**	**322**	**314**	**318**	**Informative**	**304**	**314**	**314**	**296**	**304**	**304**	**Informative**
D20S1063	202	202	202	202	202	203	Excludes paternal heterodisomy	202	202	202	202	202	202	Uninformative
D20S496	205	205	205	205	205	205	Uninformative	205	205	205	205	205	205	Uninformative
AFMB332XD5	75	75	75	75	75	75	Uninformative	75	75	75	75	75	75	Uninformative
D20S459	237	231	231	235	237	231	Excludes isodisomy	231	231	231	231	231	231	Uninformative
A/B Pentan	3	3	3	2	3	2	Excludes heterodisomy	2	3	3	3	2	3	Excludes isodisomy
SNV (c.140–53)	C	T	C	T	C	T	Excludes isodisomy	C	T	T	C	C	C	Excludes isodisomy
rs7121	C	C	C	T	C	C	Excludes maternal heterodisomy	T	T	T	C	T	C	Excludes heterodisomy
rs3730173	C	C	C	C	C	C	Uninformative	T	C	C	C	T	C	Excludes isodisomy
rs234628	T	T	T	C	T	C	Excludes heterodisomy	C	T	T	C	C	C	Excludes isodisomy
D20S443	139	139	139	135	139	139	Excludes maternal heterodisomy	139	139	139	135	139	135	Excludes heterodisomy
D20S171	**132**	**128**	**128**	**134**	**132**	**132**	**Informative**	132	138	132	138	132	138	Excludes isodisomy
AFMa202yb9	243	243	243	243	243	243	Uninformative	243	243	243	243	243	251	Excludes paternal heterodisomy
D20S173	182	184	184	182	182	182	Excludes isodisomy	184	182	182	182	184	182	Excludes isodisomy

Fluorescent PCR and genotyping results of the 27 polymorphic markers in chromosome 20q, situated in the table according to their chromosomal location. Figures indicate the obtained size of the amplicons, or the number of pentanucleotide repeats, in the case of the A/B pentanucleotide marker. In all cases, first columns indicate paternally inherited alleles; the second columns indicate maternally inherited alleles (in the cases in which alleles are not directly informative, this inheritance has been assigned assuming both paternal alleles are present; see text for details). It was not possible to determine the inheritance of the single nucleotide variation (SNV) c.140–53. Bold figures indicate informative markers.

## Results

### Structural analysis of the GNAS locus

As expected, neither patient GS0131 nor GS0155 present any point mutation along the *GNAS* gene.

### MS-MLPA

Dosage analysis of the *GNAS* locus by MS-MLPA revealed that GS0131 carried a duplication which included the 23 probes corresponding to the region from NESP55 to *TH1L* (Fig. 2A, black bars). Methylation analysis with the same technique revealed a complete alteration of the maternal methylation pattern (loss of methylation at exon A/B, XLαs and NESPAS and a gain of methylation at exon NESP55) (Fig. 2B, black bars).

The MS-MLPA study of his parents revealed that the duplication occurs ‘de novo’ in GS0131, as we could not see any alteration occurring in either parent. Both their gene dosage and their methylation pattern were normal (Fig. 2C and 2D, upper panels).

In the second family, dosage analysis of the *GNAS* locus by MS-MLPA showed that GS0155 presented with a duplication affecting 10 probes from NESP55 to XLαs, and not affecting exon A/B or the GSα promoter (Fig. 2A, grey bars). Regarding methylation, she suffered a complete loss of methylation at exon A/B, a partial gain of methylation at exons XLαs and NESPAS and a partial loss of methylation at NESP55 (Fig. 2B, grey bars).

In this case, the study of her parents revealed that GS0155 inherits the duplication from her mother, who also had 10 affected probes (Fig. 2C, lower panel, grey bars). GS0155’s mother also presents an altered methylation pattern (Fig. 2D, lower panel, grey bars), with a partial loss of methylation at exons XLαs and NESPAS (probes P9 to P15), and a partial gain at NESP55 (probes P6-P8). In this case, however, she does not have any epigenetic alteration in exon A/B (probes P16 and P17). GS0155’s father has normal dosage and methylation patterns (Fig. 2C and 2D, lower panels, black bars).

As suggested by Mantovani *et al* [2], methylation status was validated by methylation-specific PCR (data not shown). MS-PCR confirmed the observed results in GS0131, and the complete loss of methylation at exon A/B in GS0155, while partial methylation results in the family, although apparent, were a little bit more difficult to interpret. We intend to perform pyrosequencing analysis of the methylation status in both families in the future, in order to triple check the obtained results.

### Analysis of polymorphic markers

We then characterized twenty seven polymorphic markers in chromosome 20q to rule out the possibility of a uniparental disomy occurring simultaneously with any of both duplications, as it has been described for PHP-Ib [15]. Although several markers are not directly informative about their inheritance, some can individually exclude either parental isodisomies or heterodisomies, whereas the combination of all the results let us assume both paternal alleles where present in the index cases (Table 2).

### Whole genome and 20q13-ter Comparative Genomic Hybridization (CGH) arrays

In order to check whether there was any big rearrangement in the genome of these families, we performed a whole genome aCGH analysis, which showed there was no other genomic alteration in either patient or their parents, apart from the 20q duplication. Besides, analysis of the parents confirmed that the duplication occurred ‘de novo’ in GS0131 and was inherited from the mother in GS0155.

**Fig 3:**
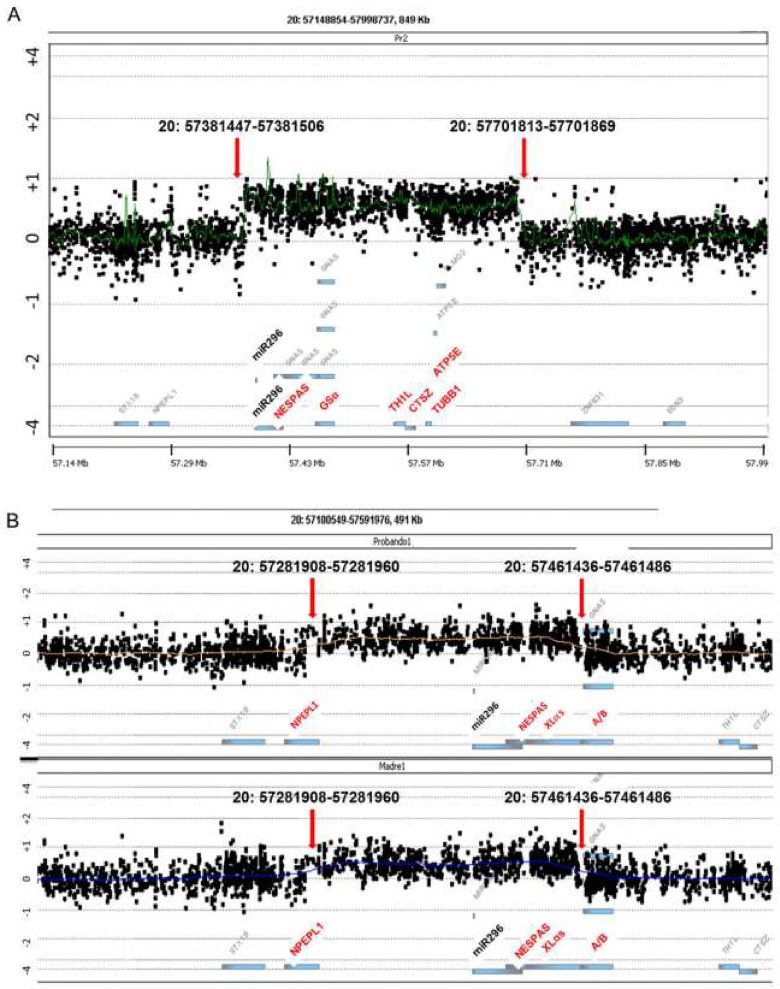
High density aCGH assay results for the 20q13 region of both families. In both cases, affected genes and regions mentioned in the text are highlighted in red on the X-axis. On the Y-axis, binary logarithm of the sample/control ratio. miR296 location is indicated in bold (corresponds to chr20:57392669–57392748). Red arrows indicate the location of the probes at each breakpoint. (A) Results for GS0131 index case. Won probes correspond to coordinates chr20:57381447–57701869. (B) Results of family GS0155 (upper panel, index case; lower panel, mother). Won probes correspond to coordinates chr20:57281908–57461486.

We then focused on the chromosomal region 20q13, using a high-density array specific only for the chromosomal coordinates chr20:47897125–62435965. Using this customized array, we were able to determine the limits of both duplications: according to hg19, GS0131 presented a ∼320 kb duplication, affecting chr20:57381447–57701869 (Fig. 3A), whereas GS0155 presented a ∼179 kb duplication, affecting chr20:57281908–57461486 (Fig. 3B, upper panel). The presence of the same duplication was confirmed in GS0155’s mother (Fig. 3B, lower panel).

### STX16 deletions

We discarded the presence in both families of the 3-kb and 4.4-kb *STX16* deletions associated with A/B isolated loss of methylation [10, 11] (data not shown).

### Determination of the parental origin of the duplication in family GS0131

**Fig 4:**
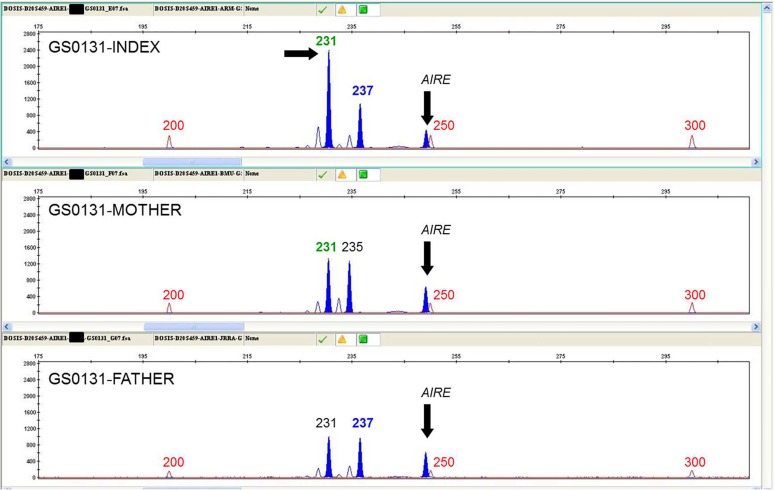
Determination of the parental origin of the duplication in family GS0131. Electropherogram showing the quantitative multiplex PCR of short fluorescent fragments (QMPSF) of microsatellite D20S459 from family GS0131 members against exon 6 of *AIRE* (21q22), used as a control amplicon. From up to down, results for the index case, mother and father. Vertical arrows show the control fragment. The horizontal arrow indicates the maternally-inherited duplicated allele in the index case. Figures show the size of the fragments in base pairs (red: weight marker; green: maternally-inherited allele; blue: paternally-inherited allele). Similar data were obtained using other control amplicons: exon 2 of *HNF1A* (12q24) and exon 3 of *HNF1B* (17q12). Data of these experiments are not shown.

In order to study the parental origin of the allele in which the duplication occurred in the GS0131 family, we carried out a QMPSF analysis of microsatellites inside the duplicated region. Only D20S459 turned out to be informative, revealing the duplication arose in the maternal allele (Fig. 4).

### Search of specific sequences

Trying to clarify the origin of both duplications, we searched within the sequences at breakpoints for different elements susceptible to being responsible for hypothetical non-allelic homologous recombination (NAHR) or non-homologous end joining (NHEJ), such as LINE or SINE elements, using RepeatMasker (http://www.repeatmasker.org/cgi-bin/WEBRepeatMasker); homologous sequences at the two ends of each duplication, using BLAST search (http://blast.ncbi.nlm.nih.gov/Blast.cgi) or DNA palindromes, using DNA-palindrome Finder (http://www.alagu-molbio.net/palin.html) [28]. We were not able to localize any significant element in any of the patients.

## Discussion

In this article we report the biggest duplications detected so far in the *GNAS* region, both present in two PHP-Ib patients and arising in the maternal allele.

We did not find any significant common element susceptible to be responsible for both rearrangements (i.e. specific sequences at the breakpoints, such as palindromes or SINE elements), so we cannot conclude whether this is a specific region prone to suffer these recombinations or both duplications are due to stochastic mechanisms.

The duplication in family GS0131 occurred ‘de novo’ in the patient. It was associated with a complete loss of methylation at the NESPAS, XLαs and A/B promoters. No other change was detected in the locus, either in the patient or in his parents. Paternal 20q disomies were also ruled out by polymorphic markers’ analysis.

This 320 kb duplication (chr20:57381447–57701869) also affected four genes located 3’ to the *GNAS* locus: *TH1L* (encoding NELFC and NELFD negative elongation factors), *CTSZ* (cathepsin Z), *TUBB1* (tubulin beta-1 chain), and *ATP5E* (the epsilon subunit of the mitochondrial ATPase F1 complex). Mutations in these genes have been associated with macrothrombocytopenia (*TUBB1*, [29]), mitochondrial complex V (ATP synthase) deficiency nuclear type (*ATP5E*, [30]) or even tumour metastasis (*CTSZ*, [31]). As far as we know, there is not any report in the literature of any duplication in this region. Furthermore, at this point, the patient does not show any sign of these disorders, so the duplication he presents only seems to clinically affect the *GNAS* locus and its phenotypic manifestations, and the clinical significance of the overdose of the rest of the genes still needs to be elucidated.

In the same way, the duplication also affects miR-296 and miR-298, two microRNAs located in the *GNAS* region (Fig. 3A). These microRNAs have been described to target *IKBKE* and other genes regulating signaling pathways [32]. Moreover, they have been shown to have an imprinted expression, as they are paternally expressed and maternally repressed. In the case of GS0131, only the “silenced” maternal allele is duplicated, so the function of both miRNAs should not be affected, although further research should be performed to check this issue.

The second duplication, present in family GS0155, appeared both in the index case and her mother. It comprised 179 kilobases (chr20:57281908–57461486) and did not affect any other gene downstream *GNAS*, although both miR-296 and miR-298 and the *NPEPL1* gene (located outside the *GNAS* locus on chr20:57264187–57294294) were also included in the affected region. In the case of the miRNAs, again, the duplicated “silenced” maternal allele is predicted not to affect their function, but this should be tested. On the other hand, *NPEPL1* codifies for the protein known as “probable aminopeptidase NPEPL1”, whose exact function has not been reported so far, but it probably catalyzes the removal of unsubstituted N-terminal amino acids from various peptides. NPEPL1 proteins might function as tumor suppressors in tumorigenesis under the control of the miR-19a microRNA, although this has also not been consistently proved [33], so the effect of the overdose of this gene also remains elusive.

Duplications and deletions affecting other imprinted loci have also been reported. For instance, ICR1 and ICR2 in 11p15, associated with Silver-Russell and Beckwith-Wiedemann syndromes [34]. The interpretation of the copy number variation in this region is complicated, because the clinical features in carriers of these alterations depend on the size, breakpoint positions and the parental inheritance of the aberrations. Some interstitial microduplications have also been reported in the Prader-Willi and Angelman critical region, in 15q11-q13, also subject to imprinting. These duplications are the most common alteration in autism. In this case, maternal duplications are associated with autism, while paternal duplications usually have a normal phenotype [35].

Regarding the *GNAS* locus, as mentioned, few genomic rearrangements have been reported, and most of them are related to PHP-Ia. In 2005, Bastepe *et al* described the up-to-date only mentioned duplication in the *GNAS* locus, present in an AD-PHP-Ib kindred [13]. In that case, the duplicated region encompassed 15kb from the region between XLαs and A/B. Furthermore, the duplication was located in place of a 4kb deletion removing NESP55 DMR and NESPAS exons 3 and 4. The authors stated that, when inherited maternally, this deletion caused the loss of methylations of the DMRs normally methylated in the maternal allele. Inherited paternally, no epigenetic alteration was observed. The phenotype observed in this patient was thus due to the disappearance of the NESP55 DMR rather than to the simultaneous duplication.

In our study, there is a maternal inheritance of the duplication in the index case in family GS0155, whereas methylation status of NESPAS, NESP55 and XLαs invites to hypothesize that there is a paternal inheritance of the duplication in her mother (the partial loss of methylation at exons XLαs and NESPAS, and partial gain at NESP55 agrees with a doubled paternal allele together with a unique maternal allele). Besides, the duplicated region should have undergone proper methylation. If this is the case, it could be speculated that only the maternal inheritance of the duplication would lead to the phenotypic manifestation of PHP-Ib, as the index’s mother is asymptomatic. Unfortunately, we did not have access to the grandparents’ samples in order to confirm this latter point. Besides, GS0155 index case also suffered a complete loss of methylation at the A/B promoter, which was not present in her mother (Fig. 2B and 2D). Therefore, we hypothesize that two different mutational events could have coincided in the index case: the first one, a duplication of a region in the locus that provoked the methylation level change, inherited from her mother. The presence of two maternal alleles would lead to the observed proportional increase in methylation levels at NESPAS and XLαs and the decrease in NESP55. The second mutation, if present, would be responsible for the isolated total loss of methylation of the A/B promoter in the patient. For this reason, we tested for the presence of the already described 3-kb and 4.4-kb *STX16* deletions associated with A/B isolated loss of methylation [10, 11] in this region, not affected by the duplication, but the analysis turned out to be negative (data not shown). We then performed a high-density cGH array analysis in the 20q region in order to be able to find any small deletion inside the duplication capable of causing an isolated loss of A/B methylation, as previously described [15] but, again, we were unable to detect any second alteration. Thus, this apparent isolated loss of A/B methylation remains unexplained. It is possible that the structure of the locus itself, together with the duplication of the maternal allele make it difficult to detect this deletion, if it exists. Or, maybe, there is another cis-acting element (or elements), still not described, controlling the methylation status of the different promoters, located in the region comprising chr20:57381447–57461486 (the common elements to both duplications) whose duplication in the maternal allele may cause a change in the methylation pattern of the region, either in a broad way, as it occurs in GS0131, or just in the A/B promoter, as it happens in GS0155.

Anyway, further studies are required in order to characterize the *GNAS* locus and the 20q region, as its imprinting pattern can be modified either by small deletions, small duplications or, as in our cases, by big duplications, which indicates that maybe there are several still unknown modifier agents which take part in the establishment or maintenance of the methylation of the different promoters in the region.
